# Association Between COVID-19 and Mortality in Hip Fracture Surgery in the National COVID Cohort Collaborative (N3C): A Retrospective Cohort Study

**DOI:** 10.5435/JAAOSGlobal-D-21-00282

**Published:** 2022-01-04

**Authors:** Eli B. Levitt, David A. Patch, Scott Mabry, Alfredo Terrero, Byron Jaeger, Melissa A. Haendel, Christopher G. Chute, Jonathan H. Quade, Brent Ponce, Steven Theiss, Clay A. Spitler, Joey P. Johnson

**Affiliations:** From the Department of Orthopaedic Surgery, University of Alabama at Birmingham, Birmingham, AL (Mr. Levitt, Dr. Patch, Dr. Mabry, Dr. Quade, Dr. Theiss, Dr. Spitler, and Dr. Johnson); the Department of Translational Medicine, Florida International University Herbert Wertheim College of Medicine, Miami, FL (Mr. Levitt and Dr. Terrero); the Department of Epidemiology, University of Alabama at Birmingham, Birmingham, AL (Dr. Jaeger); the Center for Health AI, University of Colorado Anschutz Medical Campus, Aurora, CO (Dr. Haendel); the Schools of Medicine, Public Health, and Nursing, Johns Hopkins University, Baltimore, MD (Dr. Chute); and the Department of Orthopaedics, Hughston Clinic, Columbus, GA (Dr. Ponce).

## Abstract

**Background:**

This study investigated the outcomes of coronavirus disease (COVID-19)-positive patients undergoing hip fracture surgery using a national database.

**Methods:**

This is a retrospective cohort study comparing hip fracture surgery outcomes between COVID-19 positive and negative matched cohorts from 46 sites in the United States. Patients aged 65 and older with hip fracture surgery between March 15 and December 31, 2020, were included. The main outcomes were 30-day all-cause mortality and all-cause mortality.

**Results:**

In this national study that included 3303 adults with hip fracture surgery, the 30-day mortality was 14.6% with COVID-19-positive versus 3.8% in COVID-19-negative, a notable difference. The all-cause mortality for hip fracture surgery was 27.0% in the COVID-19-positive group during the study period.

**Dicussion:**

We found higher incidence of all-cause mortality in patients with versus without diagnosis of COVID-19 after undergoing hip fracture surgery. The mortality in hip fracture surgery in this national analysis was lower than other local and regional reports. The medical community can use this information to guide the management of hip fracture patients with a diagnosis of COVID-19.

Coronavirus disease (COVID-19) is the disease caused by infection with the severe acute respiratory syndrome coronavirus 2 virus with recent estimates as of February 2021 totaling more than 110 million global confirmed cases and more than 2.4 million global deaths.^1,2^ Although several articles have been published, high-quality data regarding mortality and morbidity in orthopaedic patients with laboratory-confirmed COVID-19 is lacking. The purpose of this study is to describe the outcomes of COVID-19-positive patients undergoing hip fracture surgery using the largest national collaborative cohort in the United States.

Hip fractures are a major cause of nonelective hospital admissions and are associated with high morbidity and mortality.^3,4,5,6^ A number of articles have been published on hip surgery in COVID-19, primarily based on expert opinion or single-institution case series.^7,8,9,10^ A systematic review of hip fracture surgery in COVID-19-positive patients, published in December 2020, reported heterogeneous findings with mortality ranging from zero to 100%.^11^ Three multicenter studies were conducted in the United Kingdom.^12-14^ Narang et al^12^ reported the largest cohort of 86 COVID-19-positive patients.

The National COVID Cohort Collaborative (N3C) Data Enclave represents the largest effort to integrate and harmonize data from sites across the United States to investigate COVID-19.^15,16^ We undertook a study of 185 COVID-19-positive hip fracture surgery patients, with control subjects matched for age, sex, and race. We hypothesized that COVID-19-positive patients would have higher all-cause mortality and morbidity compared with COVID-19-negative patients. These analyses provide insight into the burden of COVID-19 in hip fracture surgery during the outset of the pandemic in the United States and can be updated as new data accrue.

## Methods, Study Design, and Populations

This retrospective cohort study represents an analysis of a national cohort with information from patients at 46 sites in the United States with surgery between March 15 and December 31, 2020. The sites that share data with N3C represent a variety of academic and community hospitals. All participants were grouped by COVID-19 reverse transcription polymerase chain reaction positive test or laboratory-confirmed negative cases.^16^ Among the total population in the national database, the COVID-19-positive group was matched to COVID-19-negative control subjects 2:1 for age, sex, and race.^16^ Participants aged 65 to 99 years were included. This study was started after approval by the Institutional Review Board (300005866).

### Diagnosis of Hip Fracture

The cohort described was determined by a group of orthopaedic surgeons using a similar method to cohorts that were previously defined.^14^ Hip fracture was identified using International Classification of Disease (ICD) 10th edition/Systematized Nomenclature of Medicine (SNOMED). Patients undergoing surgery were queried with Current Procedural Terminology (CPT) codes.^17^ The concept set included 196 SNOMED codes with linked ICD-10 (eg, S72) and 6 CPT codes (27130, 27125, 27235, 27236, 27244, 27245) with additional detail available as a Supplemental file, http://links.lww.com/JG9/A180. The patients included in the study had to have a diagnosis of hip fracture and undergo surgery. Surgery had to occur 7 days before or up to 30 days after diagnosis of COVID, consistent with the definition in the COVID Surg Collaborative study.^18^ For patients with multiple surgeries, the first surgery was considered the index operation. STROBE reporting guidelines were used.^19^

### National COVID Cohort Collaborative Database

N3C represents the community of clinicians, researchers, and data scientists who designed the centralized N3C Data Enclave to study COVID-19 and identify potential management strategies.^15,16^ The rationale and design article by Haendel et al^20^ described how N3C established a robust pipeline for electronic health record data integration on a secure Data Enclave enabled by collaborative, large-scale, computationally-intensive data science. In summary, an unprecedented need existed to integrate data from multiple sites across the United States that led to the development of processes to gather and validate information efficiently while maintaining data quality.^20^

### Analysis Variables

The primary outcomes were decided upon in reference to the literature specific to hip fracture surgery and regarding COVID-19.^14,21-23^ Baseline information was collected on demographics including age, sex, and race. Age was determined at the time of visit. Severe acute respiratory syndrome coronavirus 2 infection was diagnosed with reverse transcription polymerase chain reaction. Comorbidity information was collected using ICD-9, and ICD-10 codes mapped to SNOMED codes according to definitions by Quan et al.^24^ Specific comorbidities included congestive heart failure, myocardial infarction, diabetes mellitus, renal disease, mild liver disease, severe liver disease, pulmonary disease, peptic ulcer disease, peripheral vascular disease, rheumatic disease, stroke, cancer, and metastatic cancer.

Perioperative complications were selected from the Center for Medicare and Medicaid Services Hospital Acquired Conditions list.^25^ The following information was included in the analysis: acute kidney injury in hospital, venous thromboembolic events, length of stay, smoking status, Charlson Comorbidity Index score, body mass index, all-cause death at 30 days, and all-cause death. Death data were captured from the electronic health record. N3C reports deaths as they are received from the sites; counts of deaths from recent weeks are incomplete, reflecting delays in reporting. These counts are updated regularly for past weeks, and the counts are not considered complete until approximately a year after the deaths occur. Death data are monitored and reviewed for accuracy by a panel of experts in observational health studies.

### Statistical Methods and Software

Patient characteristics were assessed overall and by COVID status. Chi-square and *t*-tests were used to assess differences in categorical and continuous study variables, respectively. Differences in all-cause mortality were assessed with a multivariable adjusted logistic regression. An initial model included adjustment for the Charlson Comorbidity Index and COVID-19 status. A second model included adjustment for variables in the initial model plus acute kidney injury, venous thromboembolic events, and sepsis. Variables used in these nested models were selected a priori based on clinical relevance. All statistical analyses were conducted at alpha = 0.05.

The analyses were performed in the N3C Data Enclave environment using R, Python, and SQL with programming packages including NumPy, Pandas, SciPy, Scikit-learn, and Statsmodels.^26^ We used reproducible pipelines that are available to any approved user of the N3C Enclave. Consistent with N3C publication principles, values less than 20 were reported as “20 or fewer” or “zero.”^27^

## Results

**Figure 1:**
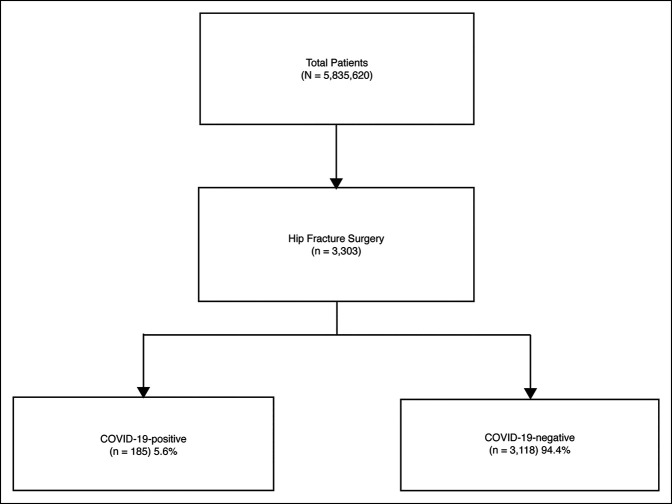
Flow diagram of study participants

A total of 3303 hip fracture surgery patients were identified in the N3C (Figure 1). Of the 3303 patients, the mean (SD) age was 82.0 (8.3) with a range between 66 and 98 years, and 67.4% were women. The study groups comprised laboratory-confirmed COVID-19-positive (5.6%) and laboratory-confirmed COVID-19-negative (94.4%). Age (82.9 ± 7.9 versus 81.9 ± 8.3, *P* = 0.09) was comparable among the two study groups with a higher proportion of women in the COVID-19-negative group (60.0% versus 67.8%, *P* = 0.03) (Table 1). Perioperative complications included acute kidney injury and sepsis. The 30-day all-cause mortality for the COVID-19-positive group was markedly higher than COVID-19-negative (14.6% versus 3.8%, *P* < 0.001). Among the COVID-19-positive group, a 27.0% (50/185) all-cause mortality existed. The all-cause mortality for the COVID-19-positive group was higher than the COVID-19-negative (27.0% versus 12.4%, *P* < 0.001).

**Table 1:** Patient Characteristics of Hip Fracture Surgery in the National COVID Cohort Collaborative

Characteristic	COVID-19-positive (n = 185)	COVID-19-negative (n = 3118)	*P*
Age,(y), (mean ± SD)	82.9 ± 7.9	81.9 ± 8.3	0.09
Sex, n (%)			0.03^a^
Female	111 (60.0)	2115 (67.8)	
Male	74 (40.0)	1003 (32.2)	
Race, n (%)			0.88
Asian	20 or fewer	48 (1.5)	
Black or African American	20 or fewer	127 (4.1)	
Pacific Islander	0 (0.0)	0 (0.0)	
Other	20 or fewer	20 or fewer	
Caucasian	160 (86.5)	2751 (88.2)	
Smoking status, n (%)			0.94
Current or former	53 (28.6)	877 (28.1)	
Non smoker	132 (71.4)	2241 (71.9)	

COVID-19 = Coronavirus disease

a Significant if *P* < 0.05.

The Charlson Comorbidity Index of the COVID-19-positive group was higher (2.4 versus 1.9, *P* = 0.008). The COVID-19-positive group had a longer length of stay (13.9 days versus 8.1 days, *P* < 0.001). Acute kidney injury was 6.9% higher in COVID-19-positive patients (23.8% versus 16.9% *P* = 0.03). Venous thromboembolic events were higher in the COVID-19-positive patients (13.5% versus 7.8%, *P* = 0.009). Additional information about perioperative information among individuals tested for COVID-19 can be found in Table 2.

**Table 2:** Clinical Information and Outcomes as No. (%) Unless Specified Otherwise

Characteristic	COVID-19-positive (n = 185)	COVID-19-negative (n = 3118)	*P*
BMI, kg/m2, (mean ± SD)	(24.7, 5.7)	(24.9, 5.4)	0.79
Length of stay, days, (mean ± SD)	(13.9, 27.0)	(8.1, 7.1)	<0.001^a^
Charlson Comorbidity Index, (mean ± SD)	(2.4, 2.8)	(1.9, 2.5)	0.008^a^
SSI	20 or fewer	29 (0.9)	0.85
Deep SSI	0 (0.0)	20 or fewer	0.93
Acute kidney injury	44 (23.8)	526 (16.9)	0.02^a^
Venous thromboembolic events	25 (13.5)	244 (7.8)	0.009^a^
Sepsis	20 (10.8)	160 (5.1)	0.002^a^

BMI = body mass index, SSI = surgical site infection

Venous thromboembolic events includes deep vein thrombosis and pulmonary embolism.

a Significant if *P* < 0.05.

In a multivariable logistic regression, COVID-19 positivity, Charlson Comorbidity Index score of three or more, acute kidney injury, and sepsis were associated with all-cause mortality (Tables 3 and 4). After adjusting for Charlson comorbidity score of 3 or more, sex, acute kidney injury, venous thromboembolic events, and sepsis, COVID-19-positive had an adjusted OR of 2.20 (95% confidence interval: 1.52 to 3.16) and Charlson score of 3 or more had an adjusted OR of 2.05 (95% confidence interval: 1.65 to 2.54) (Table 4).

**Table 3:** Unadjusted and Adjusted Odds Ratios (95% CI) for Outcome of All-Cause Death

Characteristic	Odds Ratio (95% CI)	Adjusted Odds Ratio (95% CI)	*P*
COVID-19-positive	2.62 (1.86-3.69)	2.48 (1.75-3.51)	<0.001^a^
Charlson score 3 or more	2.40 (1.95-2.95)	2.36 (1.92-2.90)	<0.001^a^

95% CI = confidence interval (LCL, 95% lower confidence limit-UCL, 95% upper confidence limit), COVID-19 = Coronavirus disease

a Significant if *P* < 0.05.

**Table 4:** Progressive Model With Unadjusted and Adjusted Odds Ratios (95% CI) for Outcome of All-cause Death

Characteristic	Odds Ratio (95% CI)	Adjusted Odds Ratio (95% CI)	*P*
COVID-19-positive	2.62 (1.86-3.69)	2.20 (1.52-3.16)	<0.001^a^
Charlson score 3 or more	2.40 (1.95-2.95)	2.05 (1.65-2.54)	<0.001^a^
Acute kidney injury	2.31 (1.84-2.91)	1.54 (1.20-1.98)	0.001^a^
Venous thromboembolic events	1.74 (1.27-2.39)	1.20 (0.85-1.69)	0.30
Sepsis	6.06 (4.43-8.30)	4.50 (3.22-6.28)	<0.001^a^

95% CI = confidence interval (LCL, 95% lower confidence limit-UCL, 95% upper confidence limit), COVID-19 = Coronavirus disease

a Significant if *P* < 0.05.

## Discussion

The COVID-19 pandemic has affected the practice of orthopaedic surgery without any US national level analysis to understand the burden of the disease on patients with hip fracture surgery. The surgical community around the world adapted to minimize the risks associated with COVID-19 by limiting elective procedures and implementing new personal protective equipment protocols with specific operational directives.^28^ Many institutions stopped elective surgery, and guidelines were developed to classify orthopaedic injuries as elective versus time-sensitive to define medically necessary orthopaedic surgery during the COVID-19 pandemic.^29^ In instances such as hip fractures, with 1-year mortality rates after surgical treatment of 20% to 30%,^4,30-32^ the outcomes of undergoing surgical intervention with COVID-19-positive status are needed for health care providers and patients to make informed decisions. Our study found a 30-day all-cause mortality rate of 14.6% (27/185) in the COVID-19-positive cohort. The all-cause mortality for COVID-19-positive patients who underwent hip fracture surgery was 27.0% (50/185) during the study period.

Two other multicenter studies on patients undergoing hip fracture surgery reported a 30.4% (7/23) and 30.5% (25/82) mortality rate within 14 and 30 days, respectively.^14,33^ Our results suggested that there is a markedly higher all-cause mortality in COVID-19-positive patients undergoing hip fracture surgery when compared with COVID-19 negative. However, the mortality in our national analysis is lower than the other multicenter studies.^14,33^ In this study, approximately one in every four patients treated for COVID-19 undergoing hip fracture surgery died during the study period. Our findings add to the literature as a novel analysis of a national-level, centralized database in the United States in this patient population.

The relevant literature on mortality and associated risk factors for developing COVID-19 in hip fracture patients is reported in a systematic review by Clement et al.^11^ Briefly, the authors found high heterogeneity across other studies in COVID-19-positive patients undergoing orthopaedic surgery with 30-day mortalities ranging from zero to 100% with a pooled rate of 35% in 21 studies.^11^ In the United Kingdom (UK), Narang et al^12^ studied thirty-day mortality after hip fracture surgery and found a relative risk of death in COVID-19-positive patients of 3.00 in a cohort of 30 patients with hip fractures and COVID-19 compared with 10 expected from the Nottingham Hip fracture score risk stratification. The adjusted odds ratio for death in our cohort of 185 patients with hip fracture surgery and COVID-19 was 2.20.

The 30-day risk of death in hip fracture surgery patients in the United States was lower than other studies, which may influence the way physicians and surgeons evaluate, manage, and counsel patients. Equally as important, this finding is helpful for patients and their families to understand the risk of COVID-19-positive hip fracture surgery. One limitation in this study is the use of all-cause death as compared with investigating specific causes of death. We do not claim that all of these patients' deaths were directly related to COVID-19 sequelae but believe that the observed mortality rate in the context of the COVID-19 pandemic is likely related to many factors including the higher Charlson Comorbidity Index in the COVID-19-positive group, potential delays to surgery, and other systemic health care factors. A strength of our study is adjusting for potential confounders with multivariable analysis. An important limitation in our study was not including time to surgery as a factor for all-cause mortality because this is an established relationship in the literature.^34^ When consulting with N3C leadership, the consensus was reached that granular time to surgery data on the magnitude of hours was not worth pursuing in the current form of the database. As the N3C database further develops, this type of analysis may be performed in the future.

We found a markedly increased proportion of COVID-19-positive individuals with acute kidney injury and sepsis. This was markedly higher than those patients who were COVID-19-negative. This finding is consistent with studies in other fields that report multiorgan effects of COVID-19.^35-37^ This information can be used by physicians and other providers to determine appropriate care pathways before and after surgical intervention for these exceedingly complex patients.

Hip fracture patients are likely on a clinical guidelines pathway that addresses the risk of deep vein thrombosis/pulmonary embolism (venous thromboembolic events [VTE]) in this population with chemoprophylaxis, with or without a COVID-19 diagnosis. Nevertheless, our study found an increased risk of VTE in COVID-19-positive patients. Owing to the known risks associated with thromboembolism and hip fracture patients, it is important that future research continues to investigate this topic.^38^ Because anticoagulation is an important treatment component of severe COVID-19, the topic of anticoagulation management in COVID-19-positive hip fracture surgery is one that necessitates further prospective studies and consensus guidelines.

Given the notable rate of mortality in those COVID-19-positive patients with hip fractures, we performed a logistic regression analysis to identify those risk factors most associated with death. COVID-19 positivity and Charlson comorbidity score of 3 or more were both markedly associated with risk of death. These findings indicate that those hip fracture patients who have systemic manifestations of COVID-19 are at the highest risk for death. This information should be used to inform discussions regarding postoperative expectations with COVID-19-positive hip fracture patients and their families.

In contrast to many recent underpowered reports regarding the impact of COVID-19 on orthopaedic injuries, this study used the centralized N3C Data Enclave with clinical information on 5.8 million patients to obtain generalizable findings. The combination of shared tools and a collaborative group enables robust research on unique research questions using a centralized data structure that includes rigorous data ingestion, harmonization, and data quality checks.

The limitations of the NC3 Database are primarily related to the emphasis of standardized clinical information that will be available for most patients, the use of all-cause death versus cause of death, and lack of time to surgery data. This causes a lack of specific orthopaedic fracture classifications, time-to-surgery, and surgical outcome information that is challenging to standardize. The analyses are based on data that are incomplete for recent weeks from some sites because of the time to ingest and harmonize the data. Finally, as with any large database study, reliance on accurate coding is a major limitation. Given that the data are compiled from many health systems using multiple CPT codes, there is the possibility of missing information and billing errors. Regarding mortality, the NC3 data are most reliable for the index hospitalization or if a patient returned to the same hospital system. This may underestimate the true mortality in the cohort.

Our study found a markedly higher mortality and morbidity in patients with hip fracture surgery who were COVID-19-positive versus those who were COVID-19-negative. The mortality in this national analysis was lower than other local and regional reports of patients with hip fracture surgery. This is likely because of these patients being at risk of systemic complications such as sepsis. The multidisciplinary teams who care for hip fracture patients can use this information to understand the medical risks related to COVID-19 in hip fracture surgery patients. Patients and their families can benefit by understanding the increased risk of hip fracture surgery with COVID-19. With the elevated risk, the medical community can guide decision-making in the care of patients with hip fractures and can counsel patients and their families about the increased risk of complications and death in hip fracture surgery for those diagnosed with COVID-19. Real-world data analysis of COVID-19 cases provides a key tool in evaluating the effects of an ongoing pandemic.
